# Brain Gray Matter Volume Is Modulated by Visual Input and Overall Learning Success but Not by Time Spent on Learning a Complex Balancing Task

**DOI:** 10.3390/jcm8010009

**Published:** 2018-12-21

**Authors:** Milos Dordevic, Marco Taubert, Patrick Müller, Jörn Kaufmann, Anita Hökelmann, Notger G. Müller

**Affiliations:** 1 German Center for Neurodegenerative Diseases (DZNE), 39120 Magdeburg, Germany; patrick.mueller@dzne.de (P.M.); notger.mueller@dzne.de (N.G.M.); 2 Chair for Training Science “Cognition and Motion”, Department Sports Science, Otto von Guericke University, 39104 Magdeburg, Germany; Marco.taubert@ovgu.de (M.T.); Anita.hoekelmann@ovgu.de (A.H.); 3 Center for Behavioral Brain Sciences (CBBS), 39106 Magdeburg, Germany; 4 Neurology Clinic, Otto von Guericke University, 39120 Magdeburg, Germany; joern.kaufmann@med.ovgu.de

**Keywords:** balance, learning, VBM, sensory-motor system, vestibular system, visual system

## Abstract

To better understand the process of neuroplasticity, this study assesses brain changes observed by voxel-based morphometry (VBM) in response to two different learning conditions. Twenty-two young, healthy subjects learned slacklining, a complex balancing task, with either their eyes open (EO, *n* = 11) or their eyes closed (EC, *n* = 11). The learning took place three times per week for four weeks, with learning periods of 1 hour, providing a total of 12 hours of learning. The scanning and testing protocols were applied at three time-points: (1) immediately before learning (baseline), (2) immediately afterwards (post-test), and (3) two months afterwards (follow-up). The EO group performed better on the task-specific test. Significant group*time interaction effects were found in sensory-motor areas at the post-test, with increases in the EO group only. The results suggest that VBM-observed brain changes in response to learning a complex balancing task vary depending on the learning success and the availability of visual input, and not solely on the amount of time spent on learning. These findings should be taken into account by future studies using similar methodologies.

## 1. Introduction

The training of balance-related activities leads to neuroanatomical changes [1] and to improvements in spatial orientation and balancing abilities [2], both of which are important for everyday life. This is true not only for healthy people, but also for people suffering from neurodegenerative disorders such as Parkinson’s disease [3]. Slackline training, for instance, causes medium-to-large task-specific improvements in balance, but also non-task-specific improvements in path integration and balancing abilities [2,4]. It has also been shown that learning how to slackline—a complex balancing activity—can lead to alterations in brain connectivity [5]. However, there is a major lack of neuroanatomical data on this topic. Our own still unpublished volumetric data obtained from a previously published study [2], revealed that intensive slackline training leads to neuroanatomical changes in related brain regions, including sensory-motor cortices. However, the results were in the expected direction of volumetric increases within the paracentral lobules only, whereas significant decreases arose in the other regions. These strong decreases in insula and subcortical areas were reported by no previous studies using structural magnetic resonance imaging (MRI) and voxel-based morphometry (VBM). Based on the recent findings of several functional MRI studies [6,7,8,9,10], which emphasized the importance of interactions between visual, vestibular, and somatosensory systems, it appeared plausible that our results came about as a consequence of this multisensory interaction. Nevertheless, such an assertion could not be experimentally proven based on the data obtained in our previous study, as our training group had been receiving inputs from all three systems during the intervention period. Electromyographic activity during balancing is not the same when eyes are open and closed, and how much a person relies on visual input depends on previous experience [11,12]. There is a large gap in knowledge about the neuroanatomical correlates of these behavioral differences. For this reason, we organized an additional study using the same intervention (i.e., slacklining training), but with visual input blocked. We hypothesized that gray matter volumetric changes in response to the two different conditions—training with open and closed eyes—will not located in the same brain regions. It is reasonable to expect that, despite the same amount of time spent on training by both groups, the participants who practice with open eyes will be more successful in learning this complex skill compared to those who practice with closed eyes. Thus, we also questioned whether eventual gray matter changes would be influenced by how well the task had been learned in a given amount of time. In other words, we also wanted to find out whether the observed brain changes were solely dependent on the amount of time spent on learning, or rather reflect learning success. 

Therefore, the aim of our study was to delineate the neuroanatomical changes that take place in response to two different conditions of learning an intensive balancing task: with and without visual input.

## 2. Experimental Section

### 2.1. Ethical Approval

This study was approved by and carried out in accordance with the recommendations of the Medical Faculty Ethics Committee at the Otto von Guericke University (approval number: 156/14). Each participant signed a document of informed consent before the beginning of the study.

### 2.2. Subjects

Eleven young (18 to 30 year old) subjects were recruited for this study and assigned to the intervention group with closed eyes (EC). The participants of the EC group were then age-matched with eleven subjects assigned to the intervention group with open eyes (EO). The two groups did not significantly differ in any of the other characteristics that were recorded. Physical activity was assessed by asking subjects how many hours (on average) they spend on sports each week. Aside from walking, all sports—including jogging, various team sports, cycling, etc.—were taken into consideration. The number of hours was recorded and summed up. Participants of both groups were paid the same amount of money for their participation in the study. Sample size and characteristics, as well as the balance-training duration were justified by our previous findings [2].

Eligible subjects for this study were all those between the ages of 18 and 30 years who had no previous experience in slacklining or similar activities (i.e., highly demanding balancing activities such as ballet dancing, rhythmic gymnastics, etc.) and normal or corrected-to-normal vision. The exclusion criteria included injuries to the musculoskeletal system and systemic diseases (e.g., cardiovascular disease, metabolic disease, nervous system diseases, etc.). Participants were recruited through advertisements in the buildings of the Otto von Guericke University in Magdeburg. 

### 2.3. Study Design

This study was planned and organized as a longitudinal study with a factorial (factors: time and group) and a within-group design (factor time within each group). The assignment of participants to groups was performed by M.D. (not involved in data collection), with all other investigators blinded to the outcome of the randomization.

The study consisted of measurements (including MRI) at three time points: baseline, one month (±2 days) after baseline (post-test), and three months after baseline (follow-up). All trainings took place in the movement lab of the German Center for Neurodegenerative Diseases from February to September 2015.

### 2.4. Intervention

The detailed intervention procedure has already been described in our previously published work [2]. Briefly, during a one-month period, the training group underwent intensive balance training consisting of 12 training units (three trainings/week with each training lasting 1 hour; maximum of two consecutive non-training days) on a 3-meter long slackline (“Power-wave 2.0” slackline rack). Whilst the EO group trained with open eyes, visual input in the EC group was prevented by asking the participants to wear a mask (Mindfold Inc., Durango, CO, USA) for the whole duration of each training session.

The trainings were led and supervised by an experienced instructor, whose assignment was to achieve the best possible skill level among the training group participants and provide verbal feedback. Each training unit consisted of a 10-minute warm up session and a 50-minute training session. The maximum group size was limited to four participants, so the instructor could dedicate enough time to each trainee. Moreover, the trainings were highly individualized, according to the skill and progression levels of each of the participants. 

The slackline tension was also individualized, so that when standing in the middle and applying a light vertical force (as during walking), the slackline would never be more than several centimeters away from the metal bar located 15 cm below. Our goal here was to keep the difficulty of the training at a higher level by maintaining the slackline slack and thus making it more unstable, rather than tight and stable—which would otherwise resemble walking on a firm surface. The length of the slackline was also intentionally set to 3 m. In this way, we wanted to achieve a higher rate of turns on the slackline, and thus a higher rate of semicircular canal stimulation. 

### 2.5. MRI

MR images were acquired on a 3 Tesla Siemens MAGNETOM Verio scanner (Syngo MR B17, Siemens, Munich, Germany) using a 32-channel head coil at three time points: baseline, post-test, and follow-up. High-resolution T1-weighted MPRAGE sequences were acquired using a 3D magnetization-prepared rapid gradient echo imaging protocol (224 sagittal slices, voxel size: 0.8 × 0.8 × 0.8 mm^3^, repetition time (TR): 2500 ms, echo time (TE): 3.47 ms, inversion time (TI): 1100 ms, flip angle: 7°).

Voxel-based morphometry (VBM) is a whole-brain unbiased technique for the analysis of regional gray matter volume and tissue changes [13] Preprocessing involved a VBM8 longitudinal pipeline tool and smoothing with a Gaussian kernel of 8 mm full width at half maximum (FWHM).

### 2.6. Behavioral Tests

Training-specific skill level was assessed through behavioral measurements of learning success as follows: Participants were asked to stand for 5 s on both legs and also on one leg each, as well as to walk on the slackline (up to a maximum of 4 lengths; 1 length = 3 m). Their performance on both standing and walking tasks were recorded at the end of the training period.

### 2.7. Outcome Variables and Data Analysis

Interaction effects in the VBM-analysis of gray matter volume changes included group and time as factors (pre-test to post-test and pre-test to follow-up for EO vs. EC contrast). In order to further investigate the within-group changes, a paired *T*-test procedure for both EO and EC groups was performed. A multiple comparison correction was applied in the form of family-wise-error (FWE) correction at voxel and cluster levels (cluster sizes are reported for each significant effect). Results were considered significant at *p* < 0.05 unless otherwise specified (uncorrected, *p* < 0.001).

The outcome variable for the neuroanatomical analysis was the structural change in brain neuroanatomy as observed by VBM. Data were analyzed with MatLab 2016a (Mathworks Inc., Natick, MA, USA) and SPM12 (UCL, London, Great Britain) with longitudinal pipeline (VBM8). Data quality control was performed by checking for artifacts and sample homogeneity using CAT12 toolbox (CAT, University Jena, Germany). 

The results of the VBM analyses were visualized using the xjView toolbox (http://www.alivelearn.net/xjview). Analysis of the behavioral data was performed with SPSS v.21 (IBM, Armonk, NY, USA).

## 3. Results

The final analysis included eleven participants in each group; their characteristics are shown in Table 1.

**Figure 1:**
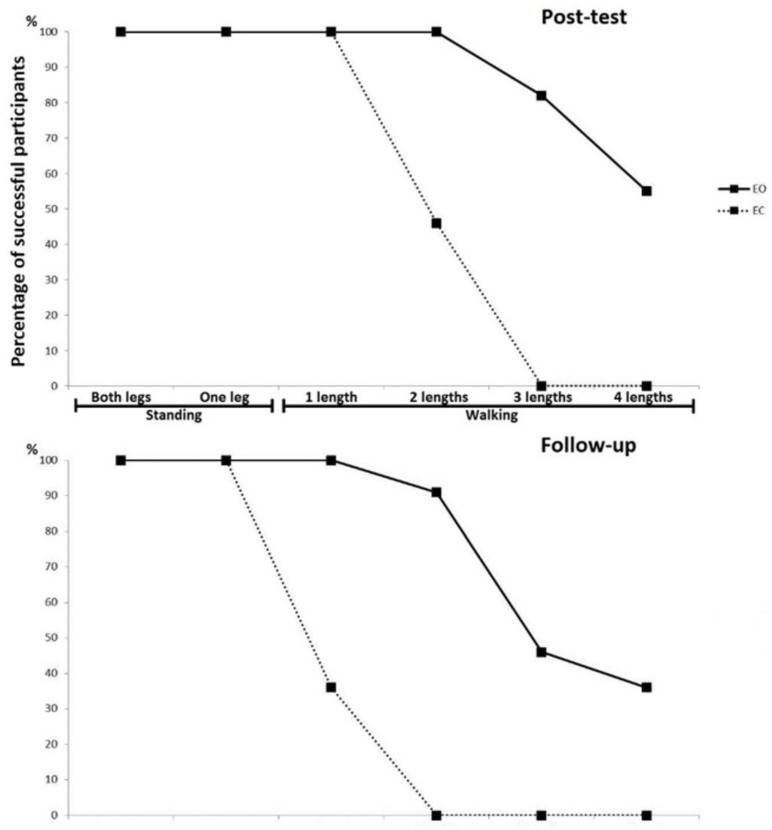
Percentage of participants who successfully achieved listed tasks at the post-test and follow-up for both groups.

Figure 1 represents specific skill learning levels (in order of difficulty) for both groups, depicting that the EO group performed much better than the EC group, both immediately after the training (post-test) and again after two months of no training (follow-up). In both groups a detraining effect could be clearly observed, whereby participants generally performed worse at the follow-up compared to their performance immediately after the training period. These achievements are also shown in Table 2. 

**Figure 2:**
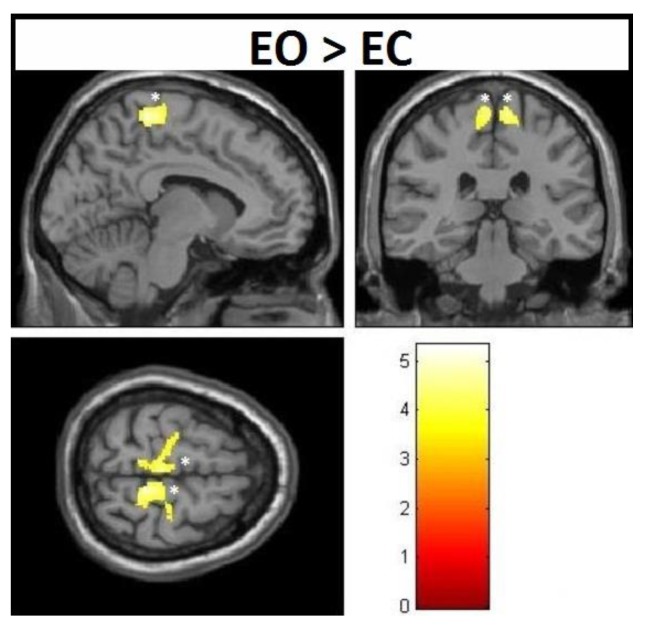
Voxel-based morphometry (VBM)-observed gray matter (GM) increments—group*time interaction effects from pre-test to post-test; * family-wise-error (FWE)-corrected.

VBM-analysis of the interaction (group*time) effects revealed significant increases at the corrected level in the EO group from pre- to post-test (Figure 2), when contrasted with the EC group. No significant interaction effects existed for other comparisons at the corrected level, neither from pre- to post-test nor from pre-test to follow-up. Montreal Neurological Institute (MNI)-coordinates, cluster sizes, and significance values of these changes are all listed in Table 3. 

**Figure 3:**
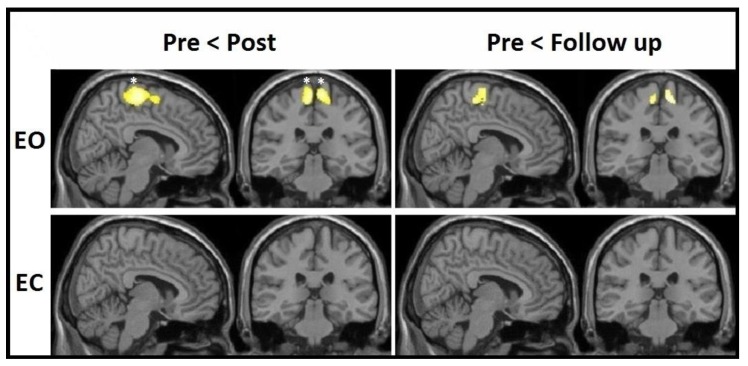
VBM-observed GM increments—within-group effects from pre-test to post-test and from pre-test to follow-up for both EO and EC groups; * FWE-corrected.

From subsequent within-group VBM analyses for both groups, it was evident that the increases in the sensory-motor cortices bilaterally were visible for the EO group only at the corrected level, with no significant effects in the EC group whatsoever (Figure 3). These corrected effects were present at the post-test, whereas a drop in both significance and cluster size could be seen at the follow-up. MNI coordinates, cluster sizes, and significance values of these changes are all listed in Table 4.

## 4. Discussion

Our results confirmed our a priori hypothesized differences in VBM-observable neuroanatomical effects between the two conditions of learning an intensive balancing task, namely with and without visual input. In addition, as also hypothesized, the group who practiced with open eyes (EO) learned much faster and achieved a much higher specific skill level during the training compared to the group who practiced with closed eyes (EC). The most striking findings were very strong transient increases in bilateral sensory-motor areas in the EO group, with no effects in these areas for the EC group whatsoever. More specifically, these changes were located in the paracentral lobules bilaterally, mainly in Brodmann’s areas 1 through 4. 

Some speculations can be made based on these results. Since increases in sensory-motor areas bilaterally existed only in the EO group, it can be assumed that visual input and/or the amount or rate of learning modulates the effects in these brain regions. From the data we have here, it is not entirely possible to discern which of the two contributed more to the observed effects. However, we assume it was the latter, since this brain region is mainly responsible for somatosensation and motor control. 

It has previously been shown that balance training causes rapid volumetric brain changes, and that these changes exhibit temporal dynamics [1,14]. However, we are not aware of any previous study that investigated differential effects of open- and closed-eye slackline training on structural brain changes, which prevents us from comparing our findings. It is known, however, that human brain functional networks have different topological organization with eyes open and eyes closed [15]. Nevertheless, our own unpublished results from a larger sample of participants (*n* = 25) who learned this skill with open eyes revealed virtually the same increase in sensory-motor areas bilaterally. The finding that the cortical regions responsible for somatosensation, movement initiation, and multisensory interaction [16,17] “enlarged” could be due to the fact that the somatosensory function becomes significantly enhanced when the vestibular system is active, as demonstrated by several studies by Ferrè et al. [6,18,19]. When both systems are active simultaneously, instead of having a supra-additive effect, the vestibular system actually becomes a modulator of a gain in somatosensation [19]. Somatosensory and motor areas were constantly receiving inputs from somatic receptors (deep and superficial) and processing them for the purpose of controlling bodily motion under these novel conditions. Thus, in the higher-order cortical sensory-motor regions, a better connectivity could have been established [20] in response to learning this new complex motor behavior, which subsequently led to their volume enlargement. However, as we did not run MRI sequences sensitive to neuronal connections (e.g., resting state functional MRI, diffusion tensor imaging) this assumption must remain speculative. 

In this study, we attempted to answer one important remaining question related to the influence of vision and multisensory interactions that involve vision on the neuroanatomical changes seen by VBM. Vision is known to be an important factor in multisensory interaction [7,8,10,21]. In the present study, the learning during training itself was not dependent on vision in the EC group, as the participants had their eyes closed. Subsequently, as indicated in the results, different effects were found for the two conditions. However, we have now arrived at another limitation which leaves us with an incomplete answer to questions regarding this matter. That is, since our training group with closed eyes did not achieve the same skill level as the EO group, we are unfortunately unable to discern how much of this difference in obtained results was due to the blocking of visual input, and how much of it was related to differences in skill levels achieved, that is, the number of lengths a participant could walk on the slackline without falling. This problem is not easy to solve, since allowing the EC group to exercise for a longer amount of time until they were able to reach the same skill level as the EO participants would have involved another variable that could have contributed to the final effects, namely the longer time spent on training. However, it appears from our data here that time spent on training itself, without significant achievement with regards to the skill level, does not contribute much to the neuroanatomical changes. Additionally, sensory-motor cortices—which underwent the largest changes—are primarily responsible for bodily sensations and movement control [22,23], thus indicating that learning success could be the dominant factor for the observed effects. Another difference between the EO and the EC groups was that the EO participants could also visually observe the other participants while they practiced. This could have driven their mirror system, which is known to become active on the observation of others’ actions. The sensorimotor areas are part of this system, and hence their volume increase in the EO group could—at least in part—have been boosted by their mirror function. However, it is unlikely that this was a major effect, as the participants were struggling to control their own movements while their vision was fixated to the wall. The necessity to focus on one’s own performance was further stressed verbally by the instructor. Hence, altogether there was little opportunity to observe others’ actions.

Besides temporal dynamics [1], another reason why the strong effects found at the post-test were completely gone at the two-month follow-up without learning could be due to a short-term stimulation from training, which lasted only one month. According to a recent meta-analysis on dose–response effects of balance trainings, for the optimal training effects one has to undergo about three months of balance training with a frequency of 3–6 trainings per week [24]. We can confirm from our data that room for further improvement still existed after our one-month intervention, even in the task-specific skills. Considering that our participants were involved in an intervention lasting less than three months, this could have possibly led to their inability to retain the transfer effects for another two months of not practicing. Erickson et al. further suggested that exercise interventions should last at least 6 to 12 months for pliable effects related to the hippocampus [25]. 

In conclusion, one month of slackline training led to different effects on participants’ brain volumetric changes in relevant areas, depending on the type of training they performed—namely, with open (EO) or closed eyes (EC). These differences between the two training conditions could particularly be seen within the sensory-motor system, where increases took place only in the EO group. The obtained brain changes in the EO group might be due to interactions of vestibular, somatosensory, and visual systems within the sensory-motor cortices that took place during training, together with the fact that the new skill was acquired well, relative to the much lower skill level achieved by the participants of the EC group. The transient nature of these strong effects, which were lost at the two-month follow-up, could potentially be explained by the temporal dynamics of such volumetric brain changes and an insufficient duration of training intervention. Whether the between-group differences in effects are predominantly caused by learning success or by the mere presence of visual input and concomitant multisensory interaction could be the topic of a future study. The findings of the current study should be taken into consideration by upcoming studies using similar methodologies.

## Figures and Tables

**Table 1:** Participant characteristics. EO: eyes-open group; EC: eyes-closed group.

Characteristic	EO (*n* = 11)	EC (*n* = 11)
Age (years)	24.1 ± 2.7	23.6 ± 3.2
Sex (females)	4 (36%)	4 (36%)
Weight (kg)	74.3 ± 11.0	73.7 ± 10.9
Height (cm)	177.8 ± 8.5	177.2 ± 8.6
Hours of activity (per week)	4.3 ± 3.5	4.5 ± 1.8
Handedness (right)	11 (100%)	11 (100%)
Profession (student)	11 (100%)	11 (100%)
Suffered a small injury (e.g., ankle sprain)	5 (36%)	4 (45%)
Ethnic origin European Asian (Indian)	11 (100%) 0 (0%)	10 (91%) 1 (9%)

**Table 2:** Percentage of participants who successfully achieved task-specific learning requirements.

		Standing		Walking			
		Both Legs	One Leg	1 Length	2 Lengths	3 Lengths	4 Lengths
Post-test	EO	100	100	100	100	82	55
	EC	100	100	100	46	0	0
Follow-up	EO	100	100	100	91	46	36
	EC	100	100	36	0	0	0

**Table 3:** MNI (Montreal Neurological Institute) coordinates (*x*, *y*, *z*) of VBM-detected gray matter interaction effects—increases over time for the EO > EC contrast; * significant at FWE-corrected level.

Contrast	Effect	Brain Region	Side	Pre-Test to Post-Test	
				No. of Voxels in Cluster (*T*-Value)	MNI Coordinates
EO > EC	Increase	Paracentral	Left	1444 (4.56 *)	−6, −29, 66; −18, −23, 69; −11, −42, 68;
			Right	1676 (5.30 *)	8, −38, 66; 18, −41, 63; 30, −23, 57;

**Table 4:** MNI coordinates (*x*, *y*, *z*) of VBM-detected gray matter increases within the EO group from pre-test to post-test; * significant at FWE-corrected level.

Group	Effect	Brain Region	Side	Post-Test		Follow-Up	
				No. of Voxels in Cluster (*T*-Value)	MNI Coordinates	No. of Voxels in Cluster (*T*-Value)	MNI Coordinates
EO	Increase	Paracentral	Left	1610 (5.21 *)	−6, −23, 63; −18, −21, 69; −14, −9, 68;	236 (4.10)	−11, −30, 59; −6, −21, 69; −9, −23, 56; −18, −21, 56;
			Right	1186 (5.16 *)	8, −32, 68; 18, −24, 63; 11, −14, 65;	408 (4.44)	11, −29, 63;
